# Retraction Note: Early or delayed cord clamping during transition of term newborns: does it make any difference in cerebral tissue oxygenation?

**DOI:** 10.1186/s13052-024-01823-6

**Published:** 2024-11-22

**Authors:** Baran Cengiz Arcagok, Hulya Bilgen, Hulya Ozdemir, Asli Memisoglu, Dilsad Save, Eren Ozek

**Affiliations:** 1 Department of Pediatrics, Division of Neonatology, School of Medicine, Acibadem University, Istanbul, Turkey; 2 Department of Pediatrics, Division of Neonatology, School of Medicine, Marmara University, Istanbul, Turkey; 3 Department of Public Health, School of Medicine, Marmara University, Istanbul, Turkey

**Retraction Note: Italian Journal of Pediatrics (2024) 50:133**

10.1186/s13052-024-01707-9

The Editor-in-Chief has retracted this article because the authors were not able to provide appropriate documentation to demonstrate that they had received ethics approval prior to undertaking the work reported.

Hulya Bilgen and Asli Memisoglu agree with this retraction. Baran Cengiz Arcagök and Eren Ozek did not explicitly state whether they agree or disagree with this retraction. The remaining authors did not respond to correspondence from the Publisher regarding this retraction.

